# Variants of genes encoding TNF receptors and ligands and proteins regulating TNF activation in familial multiple sclerosis

**DOI:** 10.1111/cns.13456

**Published:** 2020-09-20

**Authors:** Laura Torre‐Fuentes, Jordi A. Matías‐Guiu, Vanesa Pytel, Paloma Montero‐Escribano, Paolo Maietta, Sara Álvarez, Ulises Gómez‐Pinedo, Jorge Matías‐Guiu

**Affiliations:** ^1^ Laboratory of Neurobiology Institute of Neurosciences IdISSC, Hospital Clínico San Carlos Universidad Complutense de Madrid Madrid Spain; ^2^ Department of Neurology Institute of Neurosciences IdISSC, Hospital Clínico San Carlos Universidad Complutense de Madrid Madrid Spain; ^3^ Nimgenetics Madrid Spain

**Keywords:** genetics, multiple sclerosis, TNF, TNF‐α, whole‐exome sequencing

## Abstract

**Introduction:**

Numerous genetic variants have been associated with susceptibility to multiple sclerosis (MS). Variants located in genes involved in specific pathways, such as those affecting TNF‐α, can contribute to the risk of MS. The purpose of this study was to determine whether variants of these genes are associated with greater risk of MS.

**Methods:**

We used whole‐exome sequencing to study genes coding for TNF‐α receptors and ligands, and proteins promoting TNF‐α expression in 116 individuals from 19 families including at least two MS patients. We compared patients with MS, patients with other autoimmune diseases, and healthy individuals.

**Results:**

Greater polymorphism was observed in several genes in families with familial MS compared to the general population; this may reflect greater susceptibility to autoimmune diseases. Pedigree analysis also revealed that LT‐α variants rs1041981 and rs2229094 and LT‐β variant rs4647197 were associated with MS and that LT‐β variant rs4647183 was associated with other autoimmune diseases. The association between autoimmune disease and *TNFAIP2* variant rs1132339 is particularly noteworthy, as is the fact that *TNFAIP6* variant rs1046668 appears to follow a recessive inheritance pattern.

**Conclusions:**

Our findings support the idea that the risk of familial MS is associated with variants of signaling pathways, including those involving TNF‐α.

## Graphical abstract

Pathways affecting TNF‐α are involved in immune signaling. Whole‐exome sequencing in 116 members of 19 families including at least two patients with multiple sclerosis was performed. Several nonsynonymous variants in TNF‐α pathways were found. The findings support the idea that the risk of familial MS is associated with variants of TNF‐α signaling pathways.

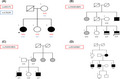

## 1 INTRODUCTION

TNF‐α receptors are transmembrane proteins that were discovered in 1984 to be associated with apoptosis, due to the death domain located on the intracellular part of the protein. To date, a total of 29 receptors and 19 ligands have been described in humans. As well as in apoptosis, this protein superfamily is involved in cell differentiation and proliferation through the activation of a range of signaling pathways, largely associated with mechanisms involved in immunity. These processes also involve proteins that promote TNF‐α expression, 10 of which have been described. On account of their involvement in immune mechanisms, many of these genes are thought to play a role in multiple sclerosis (MS). We analyze the presence of genomic variants of these genes in the families of patients with familial MS.

## 2 METHODS

### 2.1 Study sample

Our sample comprised 116 members of 19 families including at least two members diagnosed with MS according to the 2010 McDonald criteria.^1^ We studied the exomes of all individuals, establishing three groups: patients with MS, patients with other autoimmune diseases (AID) included in the American Autoimmune Related Diseases Association's list of autoimmune diseases, and healthy individuals. Families were classified as type A, in which all patients with MS belonged to the same generation, or type B, in which more than one generation was affected by MS, as described in a previous study.^2^ For all participants, we gathered data on demographic variables, personal history, clinical variables, age of onset, progression time (defined as the time from the presentation of the first neurological symptom associated with MS to the date of inclusion in the study), and clinical form of MS. This study was approved by our center's Ethics Committee. All participants included in the study gave written informed consent.

### 2.2 Whole‐exome sequencing

Blood samples were gathered from all participants. DNA was extracted using the MagNA Pure System (Roche Molecular Systems, Inc) for automated nucleic acid purification; DNA concentration and purity were evaluated with Qubit™ 2.0 and NanoDrop (Thermo Fisher Scientific, Inc), respectively. The library was prepared with the Ion AmpliSeq™ Exome Kit (Thermo Fisher Scientific, Inc), covering >97% of consensus coding sequences and adjacent splice sites (5 bp). This panel is approximately 58 Mb in size. To achieve high coverage uniformity, DNA libraries were quantified by qPCR and subsequently prepared and enriched with the Ion Chef™ System. The Ion Proton System for next‐generation sequencing (Thermo Fisher Scientific, Inc) achieved a coverage of over 90% amplicons with at least 20 reads and a mean coverage depth of >100 reads for whole‐exome sequencing. We used the Torrent Mapping Alignment Program to align the sequences obtained to the reference genome (Genome Reference Consortium human genome 19, build 37). Sequences were subsequently filtered according to specific quality criteria and analyzed using the Variant Caller tool to identify nucleotide variations as compared to the reference genome. All identified variants were annotated using the latest available version of the Ion Reporter™ software (Thermo Fisher Scientific, Inc). We aimed to identify single‐nucleotide variants and indels located within exons and splice sites of the genes analyzed, which would cause protein alterations with a minimum variant allele frequency (VAF) of 40%.

We analyzed coding regions and splice sites of genes coding for TNF‐α receptors, their ligands, and proteins promoting activation of TNF‐α proteins (with the exception of *TNF‐α*, *CD40LG*, *TNFR1*, *TNFR2*, and *TNFRSF5*, which will be analyzed elsewhere), and created a list of previously described variants (http://www.ncbi.nlm.nih.gov/SNP/, http://www.1000genomes.org, http://gnomad.broadinstitute.org, and http://evs.gs.washington.edu/EVS). The genes analyzed and the corresponding protein names, alternative names, and gene locations are listed in Table S1. For all variants, we recorded data on population frequency (minor allele frequency [MAF]) (http://gnomad.broadinstitute.org/). In order to determine the potential biological functions of the variants selected, we estimated the functional effects of the genomic variations classified as pathogenic using seven prediction algorithms (SIFT, PROVEAN, PolyPhen‐2, MutationTaster, MutationAssessor, LRT, and FATHMM) included in the ALAMUT (http://www.interactive‐biosoftware.com) and ANNOVAR analysis packages. We used the Combined Annotation Dependent Depletion tool (CADD, version 1.3; https://cadd.gs.washington.edu/) to predict damage to protein function.^3^, ^4^ According to the CADD scoring criteria, functional variants score ≥10, deleterious variants score ≥20, and disease causal variants score ≥30. Finally, we reviewed candidate genes mentioned in the literature available on PubMed and included in the Online Mendelian Inheritance in Man catalog.

### 2.3 Analysis of variants

Variants were described using the dbSNP record, when available. The results of the descriptive analysis of variants are expressed as absolute frequencies (%), means (standard deviation), or medians (interquartile range). The More Powerful Quasi‐Likelihood Score test (MQLS‐XM)^5^, ^6^ was used to test associations between patients and controls; this test is used with samples including related individuals. Allele frequencies were analyzed to test for deviations from Hardy‐Weinberg equilibrium. The Bonferroni correction for multiple comparisons was applied. We analyzed the variants detected in each group, accounting for family type. We also calculated odds ratios (OR) with 95% confidence intervals (CI). We used the chi‐square test to compare frequencies between groups where the MQLS‐XM test could not be applied. These comparisons were adjusted with the Fisher test or Yates correction when necessary (Table S2). A pedigree analysis was performed for the nonsynonymous variants using the criteria established by Sadovnick et al^7^: Variants present in healthy individuals and not observed in at least two family members with MS were considered not to segregate with disease. Bioinformatics filtering also detected possible variants not previously registered on dbSNP.

## 3 RESULTS

### 3.1 Description of the sample

Our sample of 116 participants from 19 families included 43 patients with MS (37.1%), 16 patients with AIDs other than MS (13.8%), and 57 healthy individuals (49.1%). Table 1 shows the characteristics of the cohort.

**Table 1:** Demographic and clinical characteristics of the cohort classified in cases with MS, cases with AID, and unaffected individuals

	MS N = 43 (37.1%)	AID N = 16 (13.8%)	Unaffected individuals N = 57 (49.1%)
Age	46.0 ± 10.5	58.6 ± 11.4	54.2 ± 19.0
Sex	Women: 25 (58.1%)	Women: 14 (87.5%)	Women: 34 (59.6%)
	Men: 18 (41.9%)	Men: 2 (12.5%)	Men: 23 (40.4%)
Clinical form of MS	RRMS: 34 (79.1%)	NA	NA
	SPMS: 5 (11.6%)		
	PPMS: 4 (9.3%)		
Progression time of MS (months)	218.3 ± 110.0	NA	NA
Other AIDs	9 (20.9%) (rheumatic fever, DM‐1, hypothyroidism, ulcerative colitis, uveitis, systemic lupus erythematosus, Guillain‐Barré syndrome)	Hypothyroidism, DM‐1, rheumatoid arthritis, uveitis, systemic lupus erythematosus, hyperthyroidism, and psoriasis	NA
Type of family^a^	Type A: 23 (53.5%)	Type A: 10 (62.5%)	Type A: 33 (57.9%)
	Type B: 20 (46.5%)	Type B: 6 (37.5%)	Type B: 24 (42.1%)

Abbreviations :AID, autoimmune disease; DM‐1, type I diabetes mellitus; MS, multiple sclerosis; NA, not applicable; RRMS, relapsing‐remitting MS; SPMS, secondary‐progressive MS; PPMS, primary‐progressive MS.

^a^ Type A: all patients with MS belong to the same generation. Type B: more than one generation is affected by MS.

### 3.2 Analysis of variants of TNF genes

All 164 SNPs followed Hardy‐Weinberg equilibrium, where *P*‐values below the Bonferroni‐adjusted threshold (*P* < .0005) would indicate significant deviations. MAFs were consulted in the gnomAD browser. We used the More Powerful Quasi‐Likelihood Score test (MQLS; MQLS‐XM), which accounts for relatedness between subjects using corrected variance. We observed no significant differences in allele frequencies between patients with MS and unaffected individuals. We used the chi‐square test to analyze differences between type A and type B families, applying Fisher test or Yates correction when necessary.

**Figure 1:**
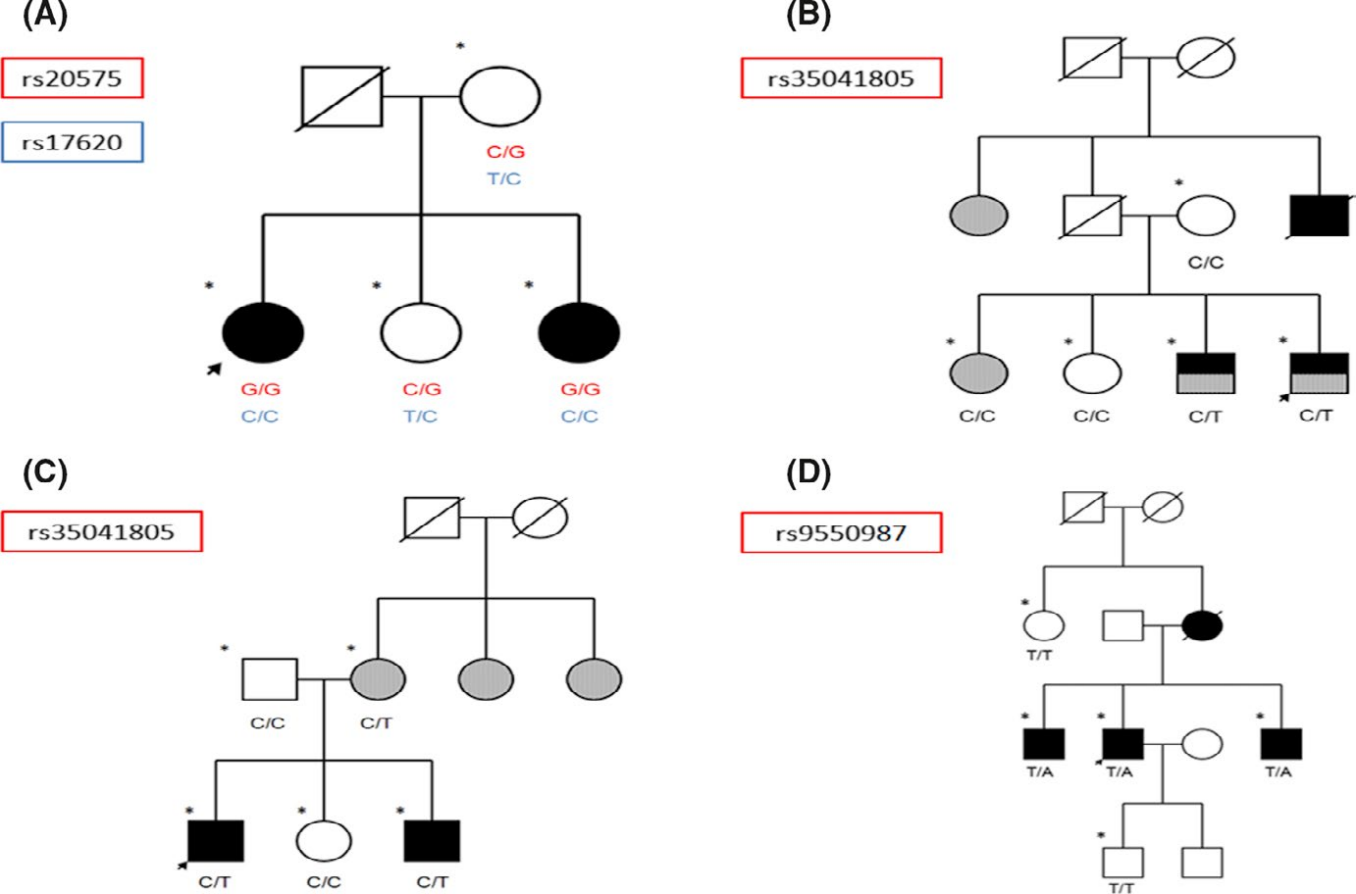
Pedigrees of families meeting criteria for establishing an association between MS and variants of genes encoding TNF receptors. A, Family with presence of variants rs20575 and rs17620 in homozygosis in individuals with MS. B, Family with presence of variant rs35041805 in individuals with MS. C, Family with presence of variant rs35041805 in individuals with MS and individuals with another AID. D, Family with presence of variant rs9550987 in individuals with MS. Arrow: proband; asterisk: whole‐exome sequencing; black: MS; gray: AID

Information on all SNPs found in our cohort is listed in Table S2. Although we did not observe significant differences in allele frequencies between patients with MS and unaffected individuals, some differences showed a trend toward significance. The rs35041805 variant of *TNFRSF19* showed greater frequency in MS cases than in individuals without the disease (*P* = .035; MQLS). This rare variant also segregated with MS and with both MS and other AIDs in two families, respectively (Figure 1).

**Figure 2:**
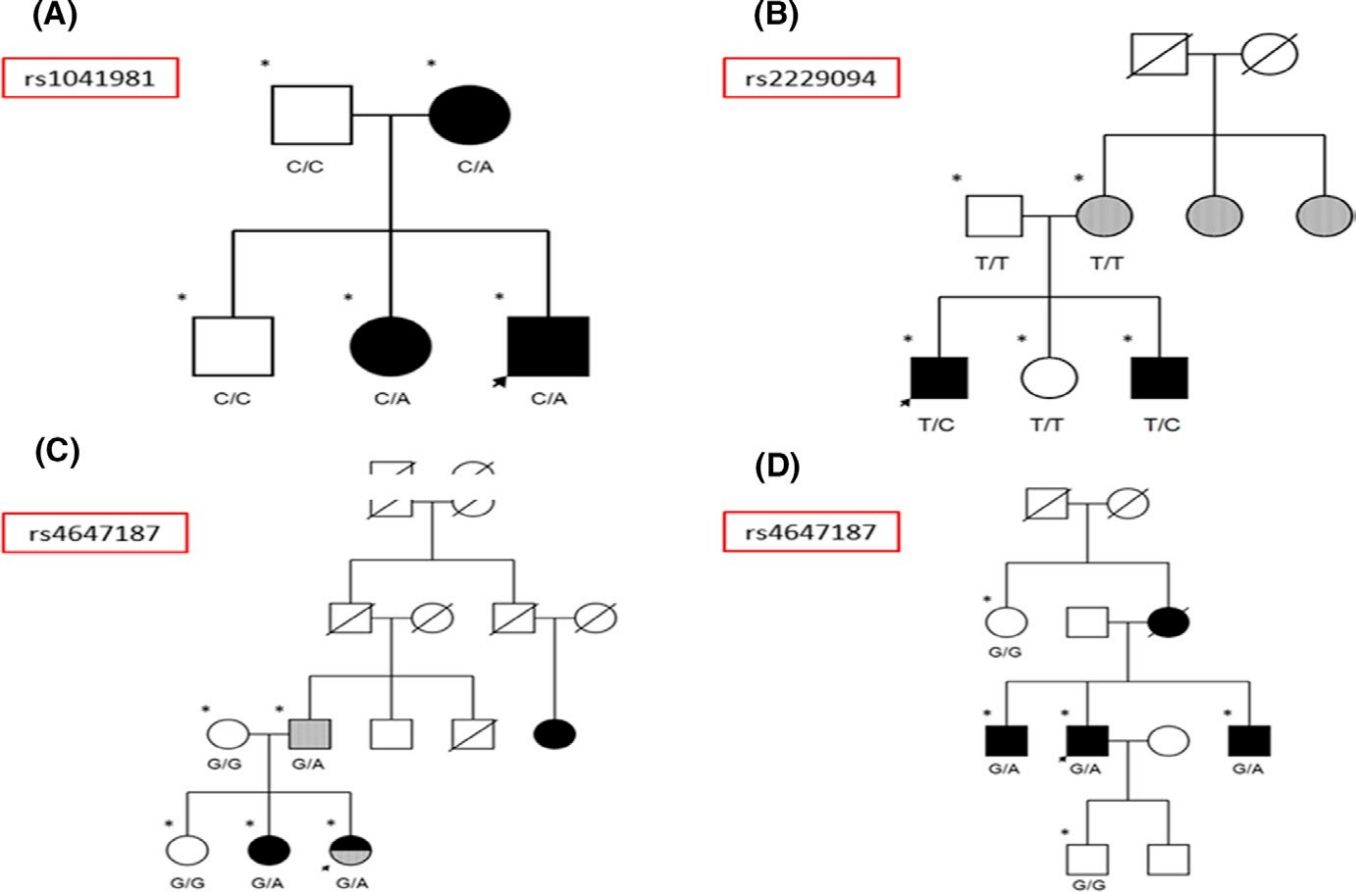
Pedigrees of families meeting criteria for establishing an association between MS and variants of genes encoding TNF ligands. A, Family with presence of variant rs1041981 in individuals with MS. B, Family with presence of variant rs2229094 in individuals with MS. C, Family with presence of variant rs4647187 in individuals with MS and individuals with another AID. D, Family with presence of variant rs4647187 in individuals with MS. Arrow: proband; asterisk: whole‐exome sequencing; black: MS; gray: AID

LT‐β variant rs4647187 was more frequent in patients with MS (*P* = .019; MQLS). While this result was not significant after correction for multiple comparisons (*P* > .0005), this variant is rare in the general population (MAF: 0.002), whereas it occurred in 6 patients with MS (14.0%), 1 patient with another AID (6.3%), and 1 healthy individual (1.8%). Moreover, rs4647187 cosegregated with MS in one family and with MS and other AIDs in another (Figure 2).

One missense variant of *TNFRSF10B*, rs1047266, was found in a higher percentage of patients with MS (14.0%) than unaffected individuals (8.2%; *P* = .013; MQLS). We also found trends to significance for variants rs2234167 of *TNFRSF14*, rs35041805 of *TNFRSF19*, and rs1046668 of *TNFAIP6*, with higher frequencies for patients with MS than for individuals without the disease.

### 3.3 Frequency of variants identified in our cohort compared to their minor allele frequency

We used the chi‐square test to analyze differences in allele frequencies between our cohort and the general population. The following variants of genes encoding TNF receptors showed greater frequencies in our cohort than the MAF: *TNFRSF10B* variant rs13265018, *TNFRSF10C* variant rs9644063, *TNFRSF10D* variant rs55636833, *TNFRS11A* variant rs35211496, *TNFRS11B* variant rs2073618, *TNFRSF19* variant rs61756242, and *EDA2R* variant rs12837393. *TNFRSF8* variant rs1763642 was much less frequent in families with MS (Table S3).

In our cohort, two variants of genes encoding TNF ligands were more frequent than would be expected from MAF data: LT‐α variant rs2229094 and *TNFSF10* variant rs16845759 (Table S3).

The variants of genes regulating TNF expression that were more frequent in our cohort were the following: *TNFAIP2* variant rs1132339, *TNFAIP3* variant rs142253225, *TNFAIP8* variant rs376335031, *TNFAIP8L3* variant rs144316469, *TNFAIP8L3* variant rs78897873, *EFNA1* variant rs4745, and *LITAF* variants rs4280262 and rs141862602 (Table S3).

### 3.4 Pedigree analysis of nonsynonymous exonic variants

**Figure 3:**
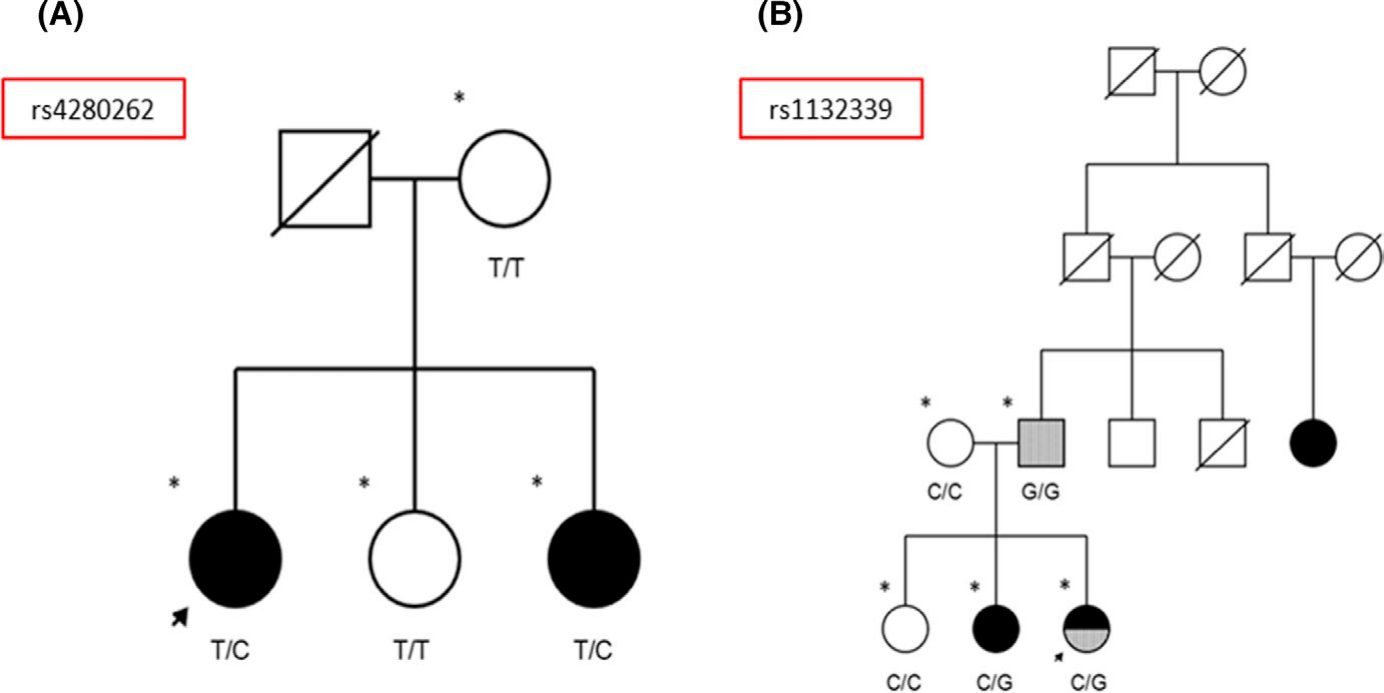
Pedigrees of families meeting criteria for establishing an association between MS and variants of genes regulating TNF expression. A, Family with presence of variant rs4280262 in individuals with MS. B, Family with presence of variant rs1132339 in individuals with MS and in homozygosis in a patient with an AID. Arrow: proband; asterisk: whole‐exome sequencing; black: MS; gray: AID

Some families met criteria for establishing an association between MS and a variant of a gene encoding a TNF receptor. The genes meeting these criteria were TNFRSF10A and TNFRSF19 (Figure 1). Furthermore, the LT‐α and LT‐β TNF ligand–coding genes (Figure 2) and the LITAF and TNFAIP2 genes, regulating TNF expression, met these criteria in the pedigree analysis (Figure 3).

### 3.5 Homozygosity of nonsynonymous exonic variants identified in the cohort

We used the chi‐square test to compare differences in the percentage of patients and controls displaying homozygosis for nonsynonymous variants. Variants of genes encoding TNF receptors identified in homozygosis were *TNFRSF7* variant rs25680; *TNFRSF8* variant rs1763642; *TNFRSF9* variant rs752649731; *TNFRSF10A* variants rs20576, rs20575, and rs17620; *TNFRSF10B* variant rs1129424; *TNFRSF10D* variants rs1133782, rs55636833, and rs11135703; *TNFRSF11A* variants rs35211496 and rs1805034; *TNFRSF11B* variant rs2073618; *TNFRSF13B* variant rs34562254; *TNFRSF14* variant rs4870; *TNFRSF14* variant rs2234167; *TNFRSF19* variant rs9550987; and *EDA2R* variant rs12837393.

Variants rs2229094 and rs1041981 of LT‐α and variant rs112120355 of *TNFSF10* were observed in homozygosis.

We also detected *EFNA1* variant rs4745, *LITAF* variant rs4280262, *TNFAIP2* variant rs1132339, *TNFAIP6* variant rs1046668, and *TNFAIP8L3* variant rs78897873 in homozygosis (Table S3).

## 4 DISCUSSION

Our study focused on one of the immune pathways related to MS. Some of these genes have previously been related to the risk of the disease.^8^ The search for genetic variations in any of these genes could lead to abnormal behavior of the pathway, potentially affecting susceptibility to MS.

Although none of the variants showed statistically significant differences in the comparison of frequencies between individuals with and without MS after correction for multiple comparisons, some variants showed a trend toward statistical significance.

We detected a rare *TNFRSF19* variant, rs35041805, which has not previously been associated with MS nor other diseases. This variant and another polymorphism of this gene, rs9550987, were segregated with the disease in two families and in one family, respectively. Previous studies have shown that this gene is downregulated in B cells from siblings with MS.^9^ TROY, the protein encoded by this gene, has also been linked to the inhibitory effects of myelin inhibitors,^10^ and its expression has been shown to be upregulated in brain tissues of patients with MS.^11^

The LT‐β gene also presented a rare variant, rs4647187, which may be associated with the disease. LT‐β, located in the 6p21.33 region, is part of the major histocompatibility complex (MHC) and has been associated with rheumatoid arthritis.^12^ We may therefore hypothesize that rs4647187 contributes to the risk of autoimmune disease in these families.

Moreover, the LT‐α gene has been linked to MS.^8^, ^13^ We identified two families displaying an association between two variants of this gene (rs1041981 and rs2229094) and MS; this association merits deeper analysis. Variant rs2229094 is particularly interesting because its frequency in the cohort was greater than we would expect given its MAF; we may therefore hypothesize that it increases the risk of autoimmune disease in these families. In addition, an increased LT‐α protein response has been reported in patients with MS, which suggests that LT‐α plays a role in the pathogenesis of the disease.^14^ Mutations in LT‐α could impact this role and the signaling pathway.

*TNFSF10* encodes the TRAIL protein, which may be involved in MS pathogenesis, according to some studies.^15^, ^16^ There are four different types of TRAIL surface receptor. The genes coding for the TRAIL receptors are *TNFRSF10A*, *TNFRSF10B*, *TNFRSF10C*, and *TNFRSF10D*. The rs1047266 polymorphism of *TNFRSF10B* was found in a higher percentage of patients with MS than individuals without the disease. This polymorphism has not previously been associated with MS. However, TRAIL has been proposed as a marker of response to treatment with interferon.^17^, ^18^ In a study of 509 patients with MS receiving this treatment, no association with interferon beta was found.^19^ While we did not observe significant differences, we detected two other variants of one of the TRAIL receptors, *TNFRSF10A*, rs20557 and rs17620, which segregated with the disease. This association should be further studied in other samples to evaluate its potential relationship with MS.

Interestingly, in one family presenting the rs1132339 variant of *TNFAIP2*, the G allele showed a clear relationship with AIDs, both in homozygosis and in heterozygosis. This variant, which has not previously been associated with disease, merits further research. Furthermore, *LITAF* polymorphism rs4280262 was detected only in patients with MS. It should also be noted that *TNFAIP6* variant rs1046668 only appeared in homozygosis in patients with MS. Our cohort also presented greater frequency of rs1046668 in patients with MS than in healthy individuals. Although this mutation has not been associated with MS, another mutation in the gene has been associated with systemic lupus erythematosus.^20^

Furthermore, we detected one *TNFRSF14* polymorphism (rs2234167) that showed a trend toward statistical significance with a higher frequency in MS cases. This polymorphism has not been associated with MS. However, *TNFSF14* and the receptor *TNFRSF14* have recently been associated with the risk of MS.^8^, ^21^ Overexpression of the membrane‐bound form in T cells leads to autoimmunity in mice. On the other hand, the soluble form of *TNFSF14* has been shown to inhibit activation. Both mRNA and protein expression of the microRNA target *TNFSF14* are reduced in patients with MS as compared to controls.^21^ *TNFRSF14* has been linked to Crohn disease and rheumatoid arthritis; it is also reported to be a herpesvirus entry mediator. A study of the gene found that the rs6684865 variant predisposed to MS, with a greater effect in patients positive for herpesvirus.^22^ This gene also shows an association with MS susceptibility, according to a study by the International Multiple Sclerosis Genetics Consortium.^8^ The above‐mentioned polymorphism should be studied in the future.

## 5 CONCLUSIONS

Genome‐wide association studies and genome studies targeting specific mutations have identified numerous genetic variants associated with susceptibility to MS, but each of these explains only a very small percentage of the risk of developing MS. However, these variants are located in genes involved in specific pathways, which suggests that risk may reside in alterations to these pathways, rather than in specific genes.^23^ It is evident that the relationship between TNF‐α, its receptors, the ligands that bind to them, and proteins that promote its activation, as well as the TNF‐α receptor–associated factors analyzed elsewhere, is associated with autoimmunity, comprising several signaling pathways involved in AIDs. This gives rise to the need to study potential associations between MS and variants of the genes encoding these proteins. We analyzed these genes in the context of familial MS, identifying several associations which merit consideration in future studies. It should be noted that we address only familial cases of MS; these genes may not represent the same risk for sporadic cases. We have not detected many variants previously associated with MS.^8^, ^24^ This may be explained by the size of our sample and a lack of statistical power. However, we found several variants with greater frequency in MS cases, suggesting a potential role in the disease. We should emphasize that greater polymorphism was observed in many genes in the families of patients with familial MS than in the general population; this may reflect greater susceptibility to autoimmune disease. Pedigree analysis also showed that the rs1041981 and rs2229094 variants of the LT‐α and rs4647197 of LT‐β genes were associated with MS and that LT‐β variant rs4647183 was associated with other autoimmune diseases. The association between *TNFAIP2* variant rs1132339 and AIDs, and the recessive inheritance pattern of *TNFAIP6* variant rs1046668 are particularly noteworthy. While these variants require further study, including functional studies analyzing their pathogenicity,^25^ these findings support the idea that the risk of familial MS may be associated with variants affecting signaling pathways, including those involving TNF‐α.

## CONFLICT OF INTEREST

The authors have no conflicts of interest to declare.

## AUTHOR CONTRIBUTIONS

JMG and JAMG were lead researchers. VP, JAMG, SA, and JMG designed the study. JAMG, PME, VP, and JMG conducted patient assessment. VP, LTF, and PME involved in family studies. VP, LTF, and UGP coordinated the data. PM performed whole‐exome sequencing. VP, JAMG, LTF, and JMG created database. VP, LTF, SA, JMG, and JAMG performed data filtering and analysis. VP, UGP, JMG, and JAMG performed statistical analysis. VP, LTF, JMG, UGP, and JAMG analyzed the results. VP, LTF, and JMG made figures and tables. JMG, VP, JAMG, and LTF coordinated the data. All authors revised the manuscript.

## ETHICS AND INFORMED CONSENT

This study was approved by the Clinical Research Ethics Committee of Hospital Clínico San Carlos. All participants gave written informed consent. Data were handled in observance of Spanish legislation regarding data protection (Organic Law 15/1999 of 13 December). Our study complies with the principles of the Declaration of Helsinki (“Recommendations guiding physicians in biomedical research involving human subjects,” Helsinki 1964, modified in October 2013).

## Supporting information

Table S1

Table S2

Table S3

## Data Availability

The datasets that support the findings of this study are available in the European Genome‐Phenome Archive repository at https://ega‐archive.org/datasets/EGAD00001005952, reference number EGAD00001005952.
