# Supplementary material for: Variants of genes encoding TNF receptors and ligands and proteins regulating TNF activation in familial multiple sclerosis
Source: CNS Neurosci Ther. 2020 Sep 20;26(11):1178–84. doi: 10.1111/cns.13456 (PMC7564193; doi:10.1111/cns.13456)
Supplement: Supplementary file 1 — Table S1 [file CNS-26-1178-s001.docx]

**Supplementary material table 1.** Genes studied in our cohort. **A)** Information of list of genes included in the study. **B)** Non-synonymous exonic variants of the genes studied in 116 individuals from 19 families including at least 2 members with MS. AID: autoimmune disease; CADD: Combined Annotation Dependent Depletion tool; MAF: minor allele frequency; MS: multiple sclerosis; ND: no data; NA: not applicable; UI: unaffected individual.

|  | **Gene** | **Description** | **Protein** | **Location** |
| --- | --- | --- | --- | --- |
| **Genes encoding TNF ligands** | *LTA* | Lymphotoxin-alpha | TNF-Beta | 6p21.33 |
|  | *TNFSF18* | Tumor necrosis factor ligand superfamily 18 | AITRL | 1q25.1 |
|  | *TNFSF9* | Tumor necrosis factor ligand superfamily 9 | CD137L | 19p13.3 |
|  | *TNFSF7* | Tumor necrosis factor ligand superfamily 7 | CD70 | 19p13.3 |
|  | *TNFSF8* | Tumor necrosis factor ligand superfamily 8 | CD30L | 9q32-33 |
|  | *TNFSF15* | Tumor necrosis factor ligand superfamily 15 | TL1 | 9q32 |
|  | *TNFSF4* | Tumor necrosis factor ligand superfamily 4 | OX40L | 1q25.1 |
|  | *TNFSF14* | Tumor necrosis factor ligand superfamily 14 | HVEML | 19p13.3 |
|  | *TNFSF13* | Tumor necrosis factor ligand superfamily 13 | APRIL | 17p13.1 |
|  | *TNFSF12* | Tumor necrosis factor ligand superfamily 12 | TWEAK | 17p13.1 |
|  | *FASLG* | Fas ligand | CD95L | 1q24.3 |
|  | *TNFSF11* | Tumor necrosis factor ligand superfamily 11 | OPGL | 13q14.11 |
|  | *TNFSF10* | Tumor necrosis factor ligand superfamily 10 | TRAIL | 3q26.31 |
|  | *LTB* | Lymphotoxin-beta | TNF- C | 6p21.33 |
|  | *TNFSF13B* | Tumor necrosis factor ligand superfamily 13b | BAFF | 13q33.3 |
|  | *EDA* | Ectodysplasin a1 | EDA1 | Xq.13.1 |
| **Genes encoding TNF receptors** | *LTBR* | Lymphotoxin B receptor | LT-βR | 12p13.31 |
|  | *TNFRSF4* | Tumor necrosis factor receptor 4 | OX40 | 1p36.33 |
|  | *TNFRSF7* | Tumor necrosis factor receptor 7 | CD27 | 12p13.31 |
|  | *TNFRSF8* | Tumor necrosis factor receptor 8 | CD30 | 1p36.22 |
|  | *TNFRSF9* | Tumor necrosis factor receptor 9 | CD137 | 1p36.23 |
|  | FAS | Fas cell surface death receptor | CD95 | 10q23.21 |
|  | *TNFRSF10A* | Tumor necrosis factor receptor 10a | TRAILR1 | 8p21.3 |
|  | *TNFRSF10B* | Tumor necrosis factor receptor 10b | TRAILR2 | 8p21.3 |
|  | *TNFRSF10C* | Tumor necrosis factor receptor 10c | TRAILR3 | 8p21.3 |
|  | *FAS (TNFRSF6B)* | Fas cell surface death receptor | FAS | 20q13.23 |
|  | *TNFRSF11B* | Tumor necrosis factor receptor superfamily, member 11b | OPG | 8q24.12 |
|  | *TNFRSF11A* | Tumor necrosis factor receptor 11a | RANK | 18q21.22 |
|  | *TNFRSF10D* | Tumor necrosis factor receptor 10d | TRAILR4 | 8p21.3 |
|  | *TNFRSF12A* | Tumor necrosis factor receptor 12a | TWEAK receptor | 16p13.3 |
|  | *TNFRSF18* | Tumor necrosis factor receptor 18 | GITR / AITR | 1p36.33 |
|  | *EDAR* | Ectodysplasin a receptor | EDA1R | 2q13 |
|  | *EDA2R* | Ectodysplasin a2 receptor | EDA-A2R | Xq12 |
|  | *TNFRSF19* | Tumor necrosis factor receptor 19 | TROY / TAJ | 13q12.12 |
|  | *RELT* | Tumor necrosis factor receptor superfamily, member 19-like | TNFRSF19L | 11q13.4 |
|  | *TNFRSF21* | Tumor necrosis factor receptor superfamily, member 21 | DR6 | 6p12.3 |
|  | TNFRSF17 | Tumor necrosis factor receptor superfamily, member 17 | BCMA | 16p13.13 |
|  | *TNFRSF13B* | Tumor necrosis factor receptor superfamily, member 13b | TACI | 17p11.2 |
|  | *TNFRSF13C* | Tumor necrosis factor receptor superfamily, member 13c | BAFFR | 22q13.12 |
|  | *TNFRSF14* | *Tumor necrosis factor receptor superfamily, member 14* | HVEM | 1p36.32 |
|  | *TNFRSF25* | *Tumor necrosis factor receptor 25* | DR3 | 1p36.31 |
| **Genes regulating TNF expression** | *LITAF* | Lipopolysaccharide-induced tumor necrosis factor-alpha factor | LPS-induced TNF-alpha factor | 16p13.13 |
|  | *TNFAIP1* | Tumor necrosis factor-alpha-induced protein 1 | TNF-alpha-induced protein 1 | 17q11.2 |
|  | *TNFAIP2* | Tumor necrosis factor-alpha-induced protein 1 | TNF-alpha-induced protein 2 | 14q32.32 |
|  | *TNFAIP3* | Tumor necrosis factor-alpha-induced protein 3 | TNF -alpha-induced protein 3 | 6q23.3 |
|  | *EFNA1 (TNFAIP4)* | Ephrin a1 | **Ephrin A1** | 1q22 |
|  | *TNFAIP6* | Tumor necrosis factor-alpha-induced protein 6 | TSG6 | 2q23.3 |
|  | *TNFAIP8* | Tumor necrosis factor-alpha-induced protein 8 | TNF Alpha Induced Protein 8 | 5q23.1 |
|  | *TNFAIP8L1* | Tumor necrosis factor-alpha-induced protein 8-like 1 | TNFAIP8-like protein 1 | 19p13.3 |
|  | *TNFAIP8L2* | Tumor necrosis factor-alpha-induced protein 8-like 2 | TNFAIP8-like protein 2 | 1q21.3 |
|  | *TNFAIP8L3* | Tumor necrosis factor-alpha-induced protein 8-like 3 | TNFAIP8-like protein 3 | 15q21.2 |
