# Supplementary material for: Variants of genes encoding TNF receptors and ligands and proteins regulating TNF activation in familial multiple sclerosis
Source: CNS Neurosci Ther. 2020 Sep 20;26(11):1178–84. doi: 10.1111/cns.13456 (PMC7564193; doi:10.1111/cns.13456)
Supplement: Supplementary file 3 — Table S3 [file CNS-26-1178-s003.docx]

**Supplementary material table 3.** A) Comparison between frequencies of variants of studied genes with frequencies in our cohort. MAF: *minor allele frequency*. CI: confidence interval. B) Presence of homozygosis in the non-synonymous exonic variants detected in the cohort. *significant differences.

**A)**

| **Genes** | **Variant** | | **MAF** | **Cohort** | **p-value**  **(Chi^2^)** |
| --- | --- | --- | --- | --- | --- |
|  |  |  |  |  |  |
| *CD27* | rs25680 | Frequency | 0.191 | 0.254 | 0.014 |
|  |  | CI (95%) | 0.189 - 0.192 | 0.202 – 0.314 |  |
| *EDA2R* | rs12837393 | Frequency | 0.002 | 0.012 | 0.016 |
|  |  | CI (95%) | 0.002 – 0.002 | 0.004 – 0.037 |  |
|  | rs1385699 | Frequency | 0.775 | 0.775 | 0.991 |
|  |  | CI (95%) | 0.773 – 0.777 | 0.718 – 0.824 |  |
| *FAS* | rs56006128* | Frequency | 0.001 | 0.017 | 0.0004 |
|  |  | CI (95%) | 0.001 - 0.001 | 0.006 – 0.043 |  |
| *TNFRSF10A* | rs2230229 | Frequency | 0.877 | 0.831 | 0.034 |
|  |  | CI (95%) | 0.876 - 0.878 | 0.778 – 0.874 |  |
|  | rs20576 | Frequency | 0.150 | 0.142 | 0.737 |
|  |  | CI (95%) | 0.148 – 0.151 | 0.103 – 0.193 |  |
|  | rs20575 | Frequency | 0.526 | 0.435 | 0.005 |
|  |  | CI (95%) | 0.524 - 0.528 | 0.373 – 0.499 |  |
|  | rs17620 | Frequency | 0.524 | 0.435 | 0.006 |
|  |  | CI (95%) | 0.522 - 0.526 | 0.373 – 0.499 |  |
| *TNFRSF10B* | rs13265018 | Frequency | 0.225 | 0.844 | 0.032 |
|  |  | CI (95%) | 0.139 - 0.344 | 0.792 – 0.885 |  |
|  | rs1129424* | Frequency | 0.680 | 0.551 | < 0.0001 |
|  |  | CI (95%) | 0.679 - 0.682 | 0.487 – 0.614 |  |
| *TNFRSF10C* | rs9644063* | Frequency | 0.294 | 0.788 | < 0.0001 |
|  |  | CI (95%) | 0.288 - 0.299 | 0.731 – 0.836 |  |
| *TNFRSF10D* | rs1133782 | Frequency | 0.653 | 0.594 | 0.062 |
|  |  | CI (95%) | 0.651 - 0.654 | 0.530 – 0.656 |  |
|  | rs55636833 | Frequency | 0.032 | 0.056 | 0.039 |
|  |  | CI (95%) | 0.031 - 0.032 | 0.033 – 0.093 |  |
|  | rs11135703* | Frequency | 0.195 | 0.051 | < 0.0001 |
|  |  | CI (95%) | 0.194 - 0.197 | 0.029 – 0.088 |  |
| *TNFRS11A* | rs35211496* | Frequency | 0.119 | 0.206 | < 0.0001 |
|  |  | CI (95%) | 0.117 - 0.120 | 0.159 – 0.263 |  |
|  | rs1805034 | Frequency | 0.546 | 0.564 | 0.577 |
|  |  | CI (95%) | 0.544 - 0.548 | 0.500 – 0.626 |  |
| *TNFRS11B* | rs2073618* | Frequency | 0.531 | 0.668 | < 0.0001 |
|  |  | CI (95%) | 0.529 - 0.533 | 0.605 – 0.725 |  |
| *TNFRSF13B* | rs56063729 | Frequency | 0.016 | 0.030 | 0.110 |
|  |  | CI (95%) | 0.015 - 0.016 | 0.014 – 0.061 |  |
| *TNFRSF13C* | rs61756766 | Frequency | 0.005 | 0.012 | 0.143 |
|  |  | CI (95%) | 0.005 - 0.005 | 0.004 – 0.037 |  |
| *TNFRSF14* | rs4870* | Frequency | 0.517 | 0.418 | 0.002 |
|  |  | CI (95%) | 0.515 - 0.519 | 0.356 – 0.482 |  |
|  | rs2234167 | Frequency | 0.126 | 0.142 | 0.477 |
|  |  | CI (95%) | 0.125 - 0.127 | 0.103 – 0.193 |  |
| *TNFRSF19* | rs9550987 | Frequency | 0.226 | 0.245 | 0.476 |
|  |  | CI (95%) | 0.224 - 0.227 | 0.194 – 0.304 |  |
|  | rs61756242 | Frequency | 0.014 | 0.025 | 0.160 |
|  |  | CI (95%) | 0.014 - 0.015 | 0.011 – 0.055 |  |
| *TNFRSF8* | rs1763642* | Frequency | 1.000 | 0.331 | 0.000 |
|  |  | CI (95%) | 0.999 - 1.000 | 0.274 – 0.394 |  |
| *LTA* | rs2229094 | Frequency | 0.267 | 0.331 | 0.025 |
|  |  | CI (95%) | 0.265 -0.268 | 0.274 – 0.394 |  |
| *TNFSF10* | rs16845759 | Frequency | 0.012 | 0.025 | 0.063 |
|  |  | CI (95%) | 0.011 – 0.012 | 0.011 – 0.055 |  |
| *EFNA1* | rs4745* | Frequency | 0.443 | 0.659 | < 0.0001 |
|  |  | CI (95%) | 0.441 - 0.445 | 0.596 – 0.717 |  |
| *LITAF* | rs4280262 | Frequency | 0.165 | 0.232 | 0.005 |
|  |  | CI (95%) | 0.163 – 0.166 | 0.183 – 0.291 |  |
|  | rs141862602* | Frequency | 0.000 | 0.021 | < 0.0001 |
|  |  | CI (95%) | 0.000 – 0.000 | 0.009 – 0.049 |  |
| *TNFAIP2* | rs1132339* | Frequency | 0.000 | 0.530 | 0.000 |
|  |  | CI (95%) | 0.000 – 0.000 | 0.466 – 0.593 |  |
| *TNFAIP3* | rs142253225 | Frequency | 0.001 | 0.012 | 0.008 |
|  |  | CI (95%) | 0.001 – 0.002 | 0.004 – 0.037 |  |
| *TNFAIP6* | rs1046668 | Frequency | 0.151 | 0.146 | 0.831 |
|  |  | CI (95%) | 0.150 - 0.152 | 0.106 – 0.197 |  |
| *TNFAIP8* | rs376335031* | Frequency | 0.000 | 0.008 | 0.0003 |
|  |  | CI (95%) | 0.000 - 0.000 | 0.002 – 0.030 |  |
| *TNFAIP8L3* | rs144316469* | Frequency | 0.000 | 0.021 | < 0.0001 |
|  |  | CI (95%) | 0.000 - 0.000 | 0.009 – 0.049 |  |
|  | rs78897873* | Frequency | 0.033 | 0.094 | < 0.0001 |
|  |  | CI (95%) | 0.033 - 0.034 | 0.063 – 0.139 |  |

**B)**

| ***Gene*** | ***Variant (rs)*** | ***Variants in homocygosis*** | ***Variants in homocygosis***  ***In MS cases*** | ***Variants in homocygosis in AID cases*** | ***Variants in homocygosis***  ***In unaffected***  ***subjects*** | **p-value**  **(Chi^2^)**  **(MS vs. AID + Unaffected individuals)** |
| --- | --- | --- | --- | --- | --- | --- |
| ***CD27*** | rs25680 | 12 | 4 | 3 | 5 | 1.000 |
| ***CD27*** | rs2532502 | 114 | 43 | 15 | 56 | 0.529 |
| ***EDA2R*** | rs1385698 | 116 | 43 | 16 | 57 | 1.000 |
| ***EDA2R*** | rs12837393 | 1 | 0 | 0 | 1 | 1.000 |
| ***EDA2R*** | rs1385699 | 74 | 31 | 9 | 34 | 0.219 |
| ***EDAR*** | rs752662417 | 0 | 0 | 0 | 0 | 1.000 |
| ***FAS*** | rs56006128 | 0 | 0 | 0 | 0 | 1.000 |
| ***RELT*** | rs12362779 | 0 | 0 | 0 | 0 | 1.000 |
| ***TNFRSF10A*** | rs2230229 | 79 | 29 | 13 | 37 | 0.920 |
| ***TNFRSF10A*** | rs20576 | 14 | 8 | 2 | 4 | 0.172 |
| ***TNFRSF10A*** | rs20575 | 24 | 6 | 3 | 15 | 0.256 |
| ***TNFRSF10A*** | rs17620 | 24 | 6 | 3 | 15 | 0.256 |
| ***TNFRSF10A*** | rs20577 | 0 | 0 | 0 | 0 | 1.000 |
| ***TNFRSF10B*** | rs13265018 | 82 | 30 | 14 | 38 | 1.000 |
| ***TNFRSF10B*** | rs1047266 | 0 | 0 | 0 | 0 | 1.000 |
| ***TNFRSF10B*** | rs1129424 | 36 | 11 | 5 | 20 | 0.442 |
| ***TNFRSF10C*** | rs746790372 | 0 | 0 | 0 | 0 | 1.000 |
| ***TNFRSF10C*** | rs61736405 | 0 | 0 | 0 | 0 | 1.000 |
| ***TNFRSF10C*** | rs9644063 | 68 | 26 | 10 | 32 | 0.920 |
| ***TNFRSF10D*** | rs1133782 | 44 | 18 | 7 | 19 | 0.639 |
| ***TNFRSF10D*** | rs55636833 | 1 | 0 | 0 | 1 | 1.000 |
| ***TNFRSF10D*** | rs11135703 | 2 | 0 | 0 | 2 | 0.529 |
| ***TNFRSF11A*** | rs35211496 | 7 | 2 | 2 | 3 | 0.711 |
| ***TNFRSF11A*** | rs1805034 | 28 | 10 | 6 | 12 | 1.000 |
| ***TNFRSF11A*** | rs61751992 | 0 | 0 | 0 | 0 | 1.000 |
| ***TNFRSF11B*** | rs140782326 | 0 | 0 | 0 | 0 | 1.000 |
| ***TNFRSF11B*** | rs2073618 | 53 | 21 | 7 | 25 | 0.740 |
| ***TNFRSF13B*** | rs34562254 | 2 | 1 | 0 | 1 | 1.000 |
| ***TNFRSF13B*** | rs56063729 | 0 | 0 | 0 | 0 | 1.000 |
| ***TNFRSF13B*** | rs34557412 | 0 | 0 | 0 | 0 | 1.000 |
| ***TNFRSF13C*** | rs61756766 | 0 | 0 | 0 | 0 | 1.000 |
| ***TNFRSF14*** | rs4870 | 15 | 6 | 2 | 7 | 1.000 |
| ***TNFRSF14*** | rs2234163 | 0 | 0 | 0 | 0 | 1.000 |
| ***TNFRSF14*** | rs2234167 | 1 | 0 | 0 | 1 | 1.000 |
| ***TNFRSF17*** | rs373496 | 116 | 43 | 16 | 57 | 1.000 |
| ***TNFRSF18*** | rs368710708 | 0 | 0 | 0 | 0 | 1.000 |
| ***TNFRSF19*** | rs9550987 | 7 | 2 | 2 | 3 | 0.711 |
| ***TNFRSF19*** | rs61756242 | 0 | 0 | 0 | 0 | 1.000 |
| ***TNFRSF19*** | rs35041805 | 0 | 0 | 0 | 0 | 1.000 |
| ***TNFRSF19*** | rs768185710 | 0 | 0 | 0 | 0 | 1.000 |
| ***TNFRSF19*** | rs3751362 | 0 | 0 | 0 | 0 | 1.000 |
| ***TNFRSF21*** | rs144939843 | 0 | 0 | 0 | 0 | 1.000 |
| ***TNFRSF25*** | rs11800462 | 0 | 0 | 0 | 0 | 1.000 |
| ***TNFRSF4*** | rs199733493 | 0 | 0 | 0 | 0 | 1.000 |
| ***TNFRSF8*** | rs1763642 | 36 | 10 | 6 | 20 | 0.236 |
| ***TNFRSF8*** | rs2230625 | 0 | 0 | 0 | 0 | 1.000 |
| ***TNFRSF8*** | rs144498730 | 0 | 0 | 0 | 0 | 1.000 |
| ***TNFRSF8*** | rs777410629 | 0 | 0 | 0 | 0 | 1.000 |
| ***TNFRSF9*** | rs752649731 | 1 | 1 | 0 | 0 | 0.370 |
| ***FASLG*** | rs530390117 | 0 | 0 | 0 | 0 | 1.000 |
| ***LTA*** | rs2229094 | 15 | 5 | 3 | 7 | 1.000 |
| ***LTA*** | rs2229092 | 0 | 0 | 0 | 0 | 1.000 |
| ***LTA*** | rs1041981 | 7 | 3 | 0 | 4 | 1.000 |
| ***LTB*** | rs4647187 | 0 | 0 | 0 | 0 | 1.000 |
| ***TNFSF10*** | rs112120355 | 3 | 1 | 0 | 2 | 1.000 |
| ***TNFSF10*** | rs16845759 | 0 | 0 | 0 | 0 | 1.000 |
| ***TNFSF11*** | rs138818878 | 0 | 0 | 0 | 0 | 1.000 |
| ***TNFSF14*** | rs344560 | 101 | 37 | 14 | 50 | 1.000 |
| ***TNFSF15*** | rs16931745 | 0 | 0 | 0 | 0 | 1.000 |
| ***EFNA1*** | rs4745 | 48 | 15 | 10 | 23 | 0.371 |
| ***LITAF*** | rs4280262 | 3 | 0 | 0 | 3 | 0.294 |
| ***LITAF*** | rs141862602 | 0 | 0 | 0 | 0 | 1.000 |
| ***TNFAIP2*** | rs1132339 | 40 | 12 | 4 | 24 | 0.345 |
| ***TNFAIP3*** | rs146534657 | 0 | 0 | 0 | 0 | 1.000 |
| ***TNFAIP3*** | rs2230926 | 0 | 0 | 0 | 0 | 1.000 |
| ***TNFAIP3*** | rs142253225 | 0 | 0 | 0 | 0 | 1.000 |
| ***TNFAIP6*** | rs75961064 | 0 | 0 | 0 | 0 | 1.000 |
| ***TNFAIP6*** | rs1046668 | 4 | 4 | 0 | 0 | 0.017 |
| ***TNFAIP8*** | rs376335031 | 0 | 0 | 0 | 0 | 1.000 |
| ***TNFAIP8L3*** | rs144316469 | 0 | 0 | 0 | 0 | 1.000 |
| ***TNFAIP8L3*** | rs78897873 | 1 | 1 | 0 | 0 | 0.370 |
